# A New Light-Emitting, Fabric-Based Device for Photodynamic Therapy of Actinic Keratosis: Protocol for a Randomized, Controlled, Multicenter, Intra-Individual, Phase II Noninferiority Study (the Phosistos Study)

**DOI:** 10.2196/12990

**Published:** 2019-04-26

**Authors:** Anne-Sophie Vignion-Dewalle, Henry Abi Rached, Elise Thecua, Fabienne Lecomte, Pascal Deleporte, Hélène Béhal, Theresa Hommel, Alain Duhamel, Rolf-Markus Szeimies, Laurent Mortier, Serge Mordon

**Affiliations:** 1 U1189 – ONCO-THAI – Image Assisted Laser Therapy for Oncology Université de Lille, INSERM, Centre Hospitalier Universitaire de Lille Lille France; 2 Department of Dermatology Centre Hospitalier Universitaire de Lille Lille France; 3 EA 2694 – Santé Publique: épidémiologie et qualité des soins, Unité de Biostatistiques Université de Lille, Centre Hospitalier Universitaire de Lille Lille France; 4 Department of Dermatology and Allergology Klinikum Vest GmbH Recklinghausen Germany

**Keywords:** photodynamic therapy, actinic keratosis, Aktilite CL 128 lamp, light-emitting fabric

## Abstract

**Background:**

Actinic keratosis (AK) is a common early in situ skin carcinoma caused by long-term sun exposure and usually develops on sun-exposed skin areas. Left untreated, AK may progress to squamous cell carcinoma. To prevent such risk, most clinicians routinely treat AK. Therapy options for AK include cryotherapy, topical treatments, curettage, excision surgery, and photodynamic therapy (PDT).

**Objective:**

The aim of this study is to assess the noninferiority, in terms of efficacy at 3 months, of a PDT protocol involving a new light-emitting device (PDT using the Phosistos protocol [P-PDT]) compared with the conventional protocol (PDT using the conventional protocol [C-PDT]) in the treatment of AK.

**Methods:**

In this randomized, controlled, multicenter, intra-individual, phase II noninferiority clinical study, subjects with AK of the forehead and scalp are treated with P-PDT on one area and with C-PDT on the contralateral area. In both areas, lesions are prepared and methyl aminolevulinate (MAL) is applied. Thirty minutes after MAL application, the P-PDT area is exposed to red light at low irradiance (1.3 mW/cm^2^) for 2.5 hours so that a light dose of 12 J/cm^2^ is achieved. In the control area (C-PDT area), a 37 J/cm^2^ red light irradiation is performed 3 hours after MAL application. Recurrent AK at 3 months is retreated. The primary end point is the lesion complete response rate at 3 months. Secondary end points include pain scores at 1 day, local tolerance at 7 days, lesion complete response rate at 6 months, cosmetic outcome at 3 and 6 months, and patient-reported quality of life and satisfaction throughout the study. A total of 45 patients needs to be recruited.

**Results:**

Clinical investigations are complete: 46 patients were treated with P-PDT on one area (n=285 AK) and with C-PDT on the contralateral area (n=285 AK). Data analysis is ongoing, and statistical results will be available in the first half of 2019.

**Conclusions:**

In case of noninferiority in efficacy and superiority in tolerability of P-PDT compared with C-PDT, P-PDT could become the treatment of choice for AK.

**Trial Registration:**

ClinicalTrials.gov NCT03076892; https://clinicaltrials.gov/ct2/show/NCT03076892 (Archived by WebCite at http://www.webcitation.org/779qqVKek)

**International Registered Report Identifier (IRRID):**

DERR1-10.2196/12990

## Introduction

### Background

Actinic keratosis (AK) is a common early in situ skin carcinoma caused by long-term sun exposure and usually develops on sun-exposed skin areas such as the face, ears, scalp, neck, forearms, and back of the hands. Left untreated, AK will progress to invasive squamous cell carcinoma (SCC) in approximately 10% of patients [1]. To reduce the risk of developing SCC, consensus guidelines recommend that clinicians routinely treat AK [2]. Treatment options include cryotherapy, topical treatments, curettage, surgical excision, and photodynamic therapy (PDT).

PDT is a cancer treatment modality combining light of appropriate wavelengths, a nontoxic photosensitizer, and sufficient molecular oxygen to generate reactive oxygen species and destroy target cells [3]. Over the last 15 years, PDT using 5-aminolevulinic acid (ALA) and PDT using methyl aminolevulinate (MAL) have been extensively investigated for the treatment of AK [4-8]. Topical application and incubation of ALA or MAL lead to selective accumulation of the endogenous photosensitizer protoporphyrin IX (PpIX) in the AK cells, and subsequent activation of PpIX by light of appropriate wavelengths induces, in the presence of oxygen, photochemical reactions leading to cell death [3].

Activation by red light using a total light dose of 37 J/cm^2^ after 3 hours of incubation with MAL is a conventional protocol, usually referred to as PDT using the conventional protocol (C-PDT), that is approved and likely the most widely used in Europe for PDT of AK [9-11]. This protocol has been reported to be an effective PDT treatment option for AK and to result in similar response rates and improved cosmetic outcomes compared with standard therapies [9]. However, high pain scores have been demonstrated with this protocol, and concurrent use of cold air analgesia may be required to prevent discomfort [12,13].

### Objectives

Recently, several protocols involving an incubation with MAL for a maximum of 30 min followed by an activation by daylight for between 1 hour 30 min and 2 hours 30 min have been investigated [14-17]. From a European consensus [18], using a 2-hour daylight activation within 30 min after MAL application leads to a protocol (photodynamic therapy using the daylight European consensus protocol [D-PDT]) as effective as and better tolerated by patients than C-PDT. This better tolerability results from the maximum of 30 min for MAL incubation and the subsequent continuous activation of small amounts of PpIX. Nonetheless, using daylight as the irradiation source is not realistic for all weather conditions [19].

New protocols designed to be as effective as C-PDT, as nearly painless as D-PDT, and usable all year round are therefore emerging. Among these alternative protocols are the Flexitheralight protocol that we have recently published [20,21] and the Phosistos protocol (PDT using the Phosistos protocol [P-PDT]) that is discussed in this study. Developed within the Phosistos project supported by the European Commission under the Competitiveness and Innovation Framework Programme (Project identifier: CIP-ICT-PSP-2013-7-621103), P-PDT uses a 30-min MAL incubation followed by 2 hours and 30 min of irradiation with a light-emitting, fabric-based device. Due to the short incubation time, P-PDT should be as nearly painless as D-PDT. Furthermore, from a recent study that discusses potential PDT overtreatment when using some protocols including C-PDT [22], P-PDT with a total light dose almost 3 times lower than that of C-PDT could prove noninferior in efficacy. Moreover, the high flexibility of the light-emitting, fabric-based device ensures an optimal irradiation of the treatment area, which is not the case with the rigid flat light sources used in C-PDT.

The aim of this randomized, controlled, multicenter, intra-individual, noninferiority study is to assess the efficacy and tolerability of P-PDT compared with those of C-PDT in treating patients with AK of the forehead and scalp.

## Methods

### Study Design

This study is a randomized, controlled, multicenter, intra-individual, noninferiority study comparing P-PDT versus C-PDT in the treatment of AK of the forehead and scalp. The study was conducted at 2 investigational sites: the department of dermatology at the Lille University Hospital in France and the Klinikum Vest in Germany.

### Study Status

Recruitment is closed and data collection is completed. Data analysis is ongoing and is expected to be completed in the first half of 2019.

### Ethical Approval

This study was performed in accordance with the ethical principles of the Declaration of Helsinki (2008) and the International Conference on Harmonization Good Clinical Practice guidelines. The study design was reviewed and approved by the French National Agency for the Safety of Medicines and Health Products (Agence Nationale de Sécurité du Médicament et des Produits de Santé; authorization number: 2016-A00010-51), the French Ethics Committee (Comités de Protection des Personnes, CPP; authorization number: CPP 03/008/2016), the Federal Institute for Drugs and Medical Devices (Bundesinstitut für Arzneimittel und Medizinprodukte; authorization number: 2015_79 1.1), and the ethics committee of the University of Münster (Ethik-Kommission der Ärztekammer Westfalen-Lippe und der Westfälischen Wilhelms-Universität; approval number: 2016-513-f-M).

### Study Population

Patients were recruited from the patient population of the investigational sites.

The inclusion and exclusion criteria for patients to be included and excluded in the study are provided in Textboxes 1 and 2, respectively.

**Inclusion criteria for patients.**

Patients were eligible to be included in the study if they met all of the following criteria:

- They had a clinical diagnosis through visual inspection and palpation of 10 to 14 previously untreated, nonpigmented, nonhyperkeratotic, grade I or II (according to the classification of Olsen et al [23]) actinic keratosis (AK) on the forehead and scalp (in case of more than 14 AK, only 14 AK were considered).
- These AK had to be distributed in 2 noncoalescing areas with a similar number and grade of AK according to the following conditions:
  - a minimum distance of 2 mm between 2 AK in the same area
  - a minimum distance of 10 mm between 2 AK, each in a different area
- Other AK treatment options were considered as unacceptable or medically less appropriate
- They did not have any AK treatment in the previous 30 days
- They are older than 18 years and affiliated to a social security system

**Exclusion criteria for patients.**

Patients were not eligible for inclusion in the study if they fulfilled 1 or more of the following criteria:

- They had a clinical diagnosis of porphyria
- They were immunosuppressed
- They used topical corticosteroids on the forehead or scalp in the previous 2 weeks
- They received local treatment (including cryotherapy; curettage and electrocoagulation; topical treatments with imiquimod, 5-fluorouracil, diclofenac, or ingenol mebutate; or photodynamic therapy) on the face or scalp in the previous 30 days
- They used topical retinoids, alpha-hydroxy acids, systemic retinoids, chemotherapy, or immunotherapy in the previous 30 days
- They had pigmented actinic keratosis
- They had known allergy to methyl aminolevulinate (MAL) or to any other ingredient of the MAL cream, peanut, or soya
- They participated within the last 30 days in other clinical studies
- They were pregnant
- They had any condition with a risk of poor protocol compliance
- They currently received regular ultraviolet radiation therapy
- They were protected by a legal regime, in emergency situations, or kept in detention

**Figure 1:**
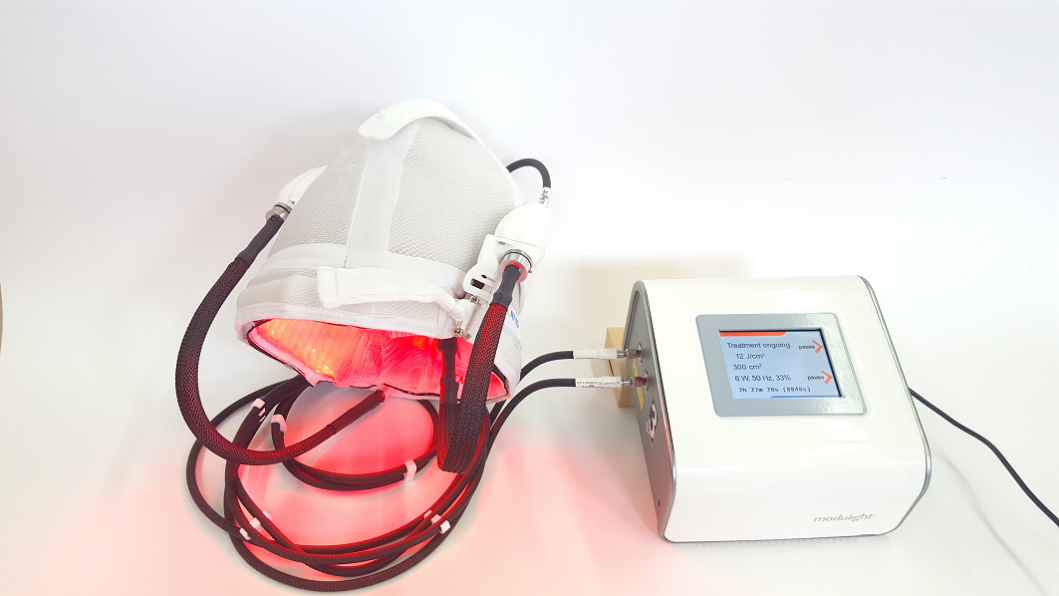
The light-emitting, fabric-based device involved in photodynamic therapy using the phosistos protocol: 635-nm red light is emitted at 1.3 mW/cm^2^ by a fiber optic–based fabric that lines the inside of a cap.

All patients received oral and written information before signing informed consent forms and subsequently entering the study.

### The Phosistos Protocol (Photodynamic Therapy Using the Phosistos Protocol [P-PDT])

P-PDT includes application of MAL cream under transparent occlusive dressing immediately followed by the installation on the patient’s head and turn-on of a light-emitting, fabric-based device for 3 hours. This device consists of a power control unit delivering 635-nm red light to a fiber optic–based fabric lining the inside of a cap (Texinov, Saint-Didier-de-la-Tour, France; Figure 1). The device, classified as exempt risk group according to IEC (International Electrotechnical Commission) 60601-2-57/2012, is configured to automatically start a 1.3mW/cm^2^ irradiation 30 min after it is turned on (resulting in an incubation time of 30 min) and to stop 2 hours and 30 min later (resulting in a light dose of 12 J/cm^2^).

### Study Objectives/Outcomes

The primary objective of the study is to assess the noninferiority, in terms of efficacy at 3 months, of P-PDT compared with C-PDT. Outcome for the primary objective is the lesion complete response rate at 3 months.

The secondary objectives are as follows:

- To evaluate the treatment tolerance including pain at the end of treatment and adverse effects at 7 days
- To evaluate the complete response rate at 6 months
- To evaluate the cosmetic results at 3 and 6 months
- To estimate the number of patients with AK reduction higher than 75% at 3 and 6 months
- To evaluate the patient’s quality of life and satisfaction throughout the study.

The corresponding outcomes are as follows:

- The pain score reported by the patient using a visual analog scale ranging from 0 (no pain) to 10 (worst pain) at the end of treatment
- The adverse effects/reactions including erythema, skin exfoliation, skin burning sensation, and skin edema reported by the patient at 7 days
- The complete response rate at 6 months
- The skin appearance (3 stands for excellent, 2 for good, 1 for fair, and 0 for poor) at 1 day, 3 months, and 6 months: the cosmetic outcome at 3 months (at 6 months) is defined as the change in skin appearance between 1 day and 3 months (6 months) and has the following possible values: –3, –2, –1, 0, 1, 2, 3
- The Dermatology Life Quality Index (DLQI) and the standard satisfaction questionnaire both completed by the patient throughout the study.

The lesion complete response (“complete response” and “incomplete response”) and the skin appearance were clinically assessed by the investigators.

### Study Schema

The study flowchart is shown in Table 1. After screening, patients entering the study had to come to the investigational site for 1 treatment visit (V1) and 3 evaluation visits (V2, V3, and V4). In case of recurrent AK at the 3-month evaluation visit (V3), patients had a second treatment visit within the 3 following weeks (V3bis).

**Table 1:** Study flowchart.

Time point	From day 30 to day 1	Day 1	Day 7±1 day	Months 3±7 days	Day 111±7 days	Months 6±7 days
Visit denomination	Screening	V1	V2	V3	V3bis in case of recurrent AK^a^	V4
Visit type	Screening	Treatment	Evaluation	Evaluation	Treatment	Evaluation
Informed consent	✓^b^	—^c^	—	—	—	—
Medical history	✓	—	—	—	—	—
Check of inclusion and exclusion criteria	✓	—	—	—	—	—
Documentation of AK including location, number, and grade	—	✓	—	✓	✓	✓
Photo-documentation of AK	—	✓	✓	✓	✓	✓
Separation of AK in 2 areas	—	✓	—	—	—	—
Randomization	—	✓	—	—	—	—
Pain score during treatment	—	✓	—	—	✓	—
Adverse effects/reactions	—	—	✓	—	—	—
Skin appearance/cosmetic outcome	—	—	—	✓	—	✓
Completion of the Dermatology Life Quality Index	—	✓	✓	✓	✓	✓
Completion of the satisfaction questionnaire	—	✓	✓	✓	✓	✓
Documentation of adverse events and serious adverse events	—	✓	—	✓	✓	✓
Pregnancy test	✓	✓	✓	✓	✓	✓

^a^AK: actinic keratosis.

^b^Indicates during which visits the actions reported in the first column were performed.

^c^Indicates that the corresponding action was not performed at the considered visit.

On the day of treatment (V1), 10 to 14 AK were located, graded, photographed, and divided into 2 areas (area A and area B) similar to each other in terms of number and grade of AK. The location of each AK was marked on plastic sheets. Randomization was then performed by opening the next envelope in sequence. This envelope specified the protocol that each area had to receive: either P-PDT for area A and C-PDT for area B or C-PDT for area A and P-PDT for area B. In both cases, P-PDT (30-min MAL incubation followed by 2.5 hours of irradiation) was performed first, so that the 3-hour MAL incubation required for C-PDT was achieved after P-PDT was completed.

Both the areas were prepared by removing crusts, gently scraping the lesion surface, and applying MAL cream (Metvixia, Galderma, France) under a transparent occlusive dressing (Tegaderm, 3M, London Ontario, Canada) to the AK and surrounding normal skin (5-10 mm margin). An aluminum foil was placed over the transparent occlusive dressing, which covered the area randomized to receive C-PDT. The device involved in P-PDT was immediately set up and turned on. After 3 hours, P-PDT was completed as described in the Phosistos Protocol section. The device involved in P-PDT was removed, and the MAL cream was washed off with saline solution. The patient rated his pain on a pain scale. The area that just received P-PDT was then protected with aluminum foil, whereas an Aktilite CL128 lamp (Galderma SA, Lausanne, Switzerland) was placed 5 to 8 cm away from the other area and programmed to deliver 37 J/cm^2^ in 7 to 10 min. At the end of C-PDT, the corresponding pain level was rated by the patient, who also completed the DLQI and the satisfaction questionnaire.

A total of 7 days after the treatment day (V2), patients were invited to report adverse effects/reactions and to complete the DLQI and the satisfaction questionnaire.

The treatment response was assessed 3 months after the treatment (V3) by the investigators by comparison with the photographs at the treatment day (V1). An investigator’s assessment of the skin appearance followed by the determination of the resulting cosmetic outcome was also performed. The DLQI and the satisfaction questionnaire were completed by patients. In case of recurrent AK, these latter were counted, graded, and photographed, and a second treatment visit, identical to the above-described first treatment (V1), was scheduled within 3 weeks after V3 (V3bis).

The last follow-up visit (V4) was performed 6 months after V1. During this visit, the treatment response and the cosmetic outcome were investigator-assessed by comparison with the photographs and the skin appearance at V1, respectively. The patients were asked to complete the DLQI and the satisfaction questionnaire.

Note that any AK appearing between V1 and V4 was not included in the assessment of the study outcomes.

### Randomization

Patients were randomly allocated to 1 of the 2 treatment options (either P-PDT for area A and C-PDT for area B or C-PDT for area A and P-PDT for area B) in a 1:1 ratio. The randomization sequence with stratification by treatment center in blocks of 4 was generated by an independent statistician using the PROC PLAN procedure of SAS (SAS Institute Inc, Cary, North Carolina, USA) and transferred to a sequence of sealed, opaque, and consecutively numbered envelopes. When a patient entered the study, randomization was performed by opening the next envelope in sequence.

The study is unblinded; both investigators and patients are aware of the treatment allocation.

### Statistical Methodology

#### Study Hypothesis

The study primary hypothesis is the noninferiority of P-PDT compared with C-PDT in terms of the lesion complete response rate at 3 months.

#### Sample Size Determination

The study was designed to have a statistical power of 80% with a 1-sided alpha level of .05 to demonstrate noninferiority in terms of lesion complete response rate at 3 months of P-PDT compared with C-PDT. Assuming a lesion complete response rate at 3 months of 75% in both areas, a correlation between lesions within the same patient, a correlation between lesions within the same area, an absolute noninferiority margin of –10%, a mean lesion number per patient per area of 6, and a possible sample loss of 10%, 270 lesions per area (ie, 45 patients) are required.

#### Statistical Analysis of the Primary Outcome

Continuous variables will be expressed as mean and SD, and categorical variables will be expressed as frequency and percentage. The normality of distribution will be assessed graphically and using the Shapiro-Wilk test.

The lesion complete response rate at 3 months will be analyzed according to the protocol using the generalized linear mixed model to take into account the patient cluster effect (a correlation between the complete responses of lesions within a same patient may exist), with adjustment on the area period (all lesions within a same area will receive the same protocol). The 1-sided 95% CI of the absolute difference in lesion complete response rate at 3 months between the 2 protocols will be calculated (D=P-PDT−C-PDT). In case of a lower limit of the 1-sided 95% CI higher than –10%, P-PDT will be declared noninferior to C-PDT and a 2-sided superiority test will be performed at an alpha level of 5%.

All statistical analyses will be performed using SAS software version 9.4 (SAS Institute Inc, Cary, North Carolina, USA).

#### Statistical Analysis of the Secondary Outcomes

The lesion complete response rate at 6 months will be processed using the same statistical analysis as the lesion complete response rate at 3 months (previous paragraph). The differences in pain scores at the end of treatment between P-PDT and C-PDT will be assessed using a linear mixed model, with patients as random effects (the significance level will be set at a 2-sided alpha level of 5%). The cosmetic outcomes at 3 and 6 months and the DLQI scores throughout the study will be compared between C-PDT and P-PDT using the Wilcoxon signed-rank test.

### Data Management

All patient data were collected using an electronic case report form according to Good Clinical Practice and Standard Operating Procedures. Data collection was regularly monitored by a clinical research associate. Any deviation from the protocol was noted and the reason for the deviation documented. Any data inconsistency was brought to the attention of the clinical team and investigational site personnel (if required, data queries were sent). Resolutions to these data inconsistencies were reflected in the database.

## Results

### Population Study

**Figure 2:**
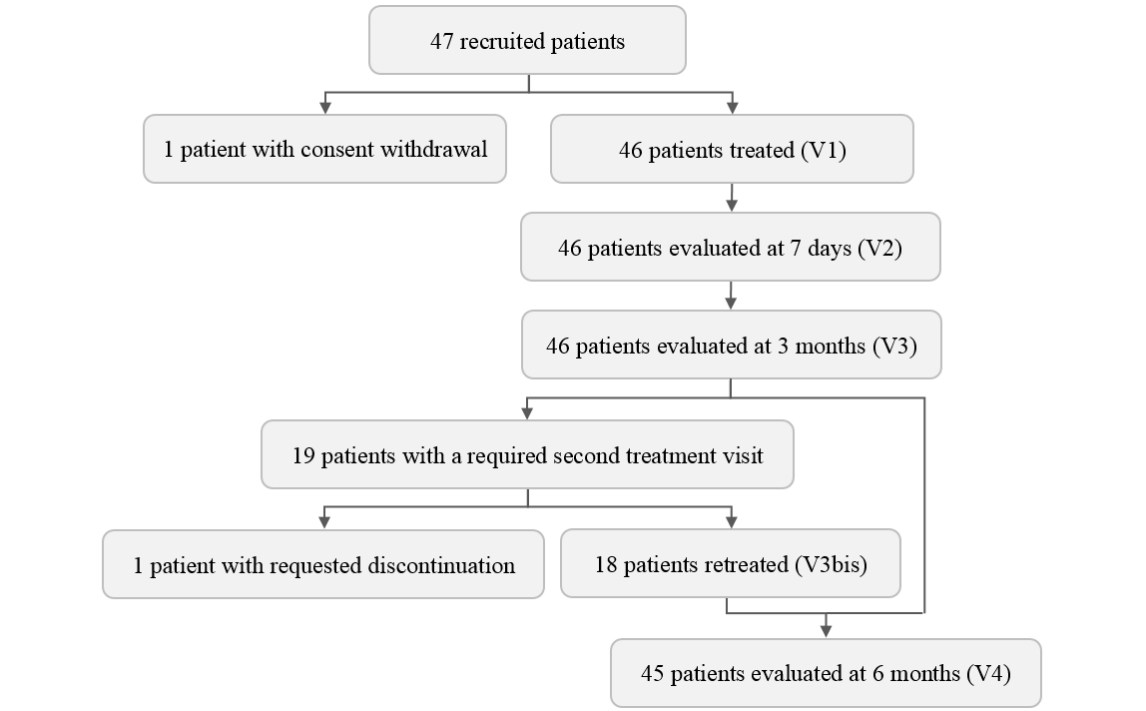
Study flow diagram. V1: first treatment visit; V2: first evaluation visit; V3: second treatment visit; V3bis in case of recurrent actinic keratosis: second treatment visit; V4: third treatment visit.

The recruitment is closed and the clinical investigations are complete (Figure 2). Of the 47 recruited patients, 1 withdrew consent and did not receive any treatment protocol. A total of 46 patients were, therefore, treated with P-PDT on 1 area (for a total of 285 AK) and with C-PDT on the contralateral area (for a total of 285 AK). All these patients were evaluated at 3 months. Due to recurrent AK, 19 patients were required to undergo a second treatment visit. One of these patients dropped out because of fear of pain as intense as that experienced with C-PDT during the first treatment visit. As a result, 18 patients were retreated and 45 patients completed the study at 6 months.

All treated patients were men, and their mean age was 72.2 years. A total of 63% (29/46) of these patients had a Fitzpatrick skin type of II (Table 2). Whatever the protocol, approximately 45% of the AK were grade I and 55% were grade II (Table 3).

**Table 2:** Demographics and clinical characteristics of the 46 treated patients.

Patients characteristics		Values
Age (years), mean (SD)		72.2 (9.1)
**Sex, n (%)**		
	Male	46 (100)
	Female	0 (0)
**Fitzpatrick skin phototype, n (%)**		
	I	8 (17)
	II	29 (63)
	III	8 (17)
	IV	1 (2)

**Table 3:** Lesions characteristics according to the protocol applied.

Lesions characteristics		Photodynamic therapy using the conventional protocol (N=285 AK^a^), n (%)	Photodynamic therapy using the Phosistos protocol (N=285 AK), n (%)
**Grade of lesions**				
	Grade I	130 (45.6)	128 (44.9)
	Grade II	155 (54.4)	157 (55.1)

^a^AK: actinic keratosis.

### Data Analysis

Data analysis is ongoing, and statistical results are expected to be available in the first half of 2019.

## Discussion

C-PDT that has been proven to be effective in many studies [9-11] is likely the most widely used approved protocol in Europe for PDT of AK. The major adverse effect of C-PDT is pain during treatment, which has been described as a burning and stinging sensation localized to the treatment area [24-26].

Several studies have recently shown that the Europe-approved D-PDT is as effective as C-PDT but better tolerated and nearly painless [18,27]. This painless characteristic comes from the short MAL incubation, which results in a continuous activation of small amounts of PpIX. Unfortunately, PDT using daylight activation depends on weather conditions [19] and cannot be performed in rainy, windy, or cold conditions unless a greenhouse is used [28]. Moreover, because of the varying intensity of daylight depending on the weather conditions and the locations, it is impossible to control the light dose.

New PDT protocols including the Flexitheralight protocol [20,21] have been designed to be as effective as C-PDT, as nearly painless as D-PDT, usable all year round, and associated with a known light dose. Consisting of a 30-min incubation with MAL followed by 2.5 hours of activation with a quite cumbersome, light-emitting, fabric-based device, which delivers 37 J/cm^2^ at an irradiance of 12.3 mW/cm^2^, the Flexitheralight protocol has been shown to be noninferior to C-PDT while being nearly pain-free [21]. We have revised downward the irradiation parameters of the Flexitheralight protocol: the new version of the Flexitheralight protocol, referred to as P-PDT, involves an irradiance of 1.3 mW/cm^2^ and a light dose of 12 J/cm^2^. The choice of such a light dose was based on a study that demonstrated the ability of 2 light sources with light doses lower than 15 J/cm^2^ to completely photobleach PpIX [28]. Regarding the irradiance, the value of 1.3 mW/cm^2^ was selected based on the study by Ibbotson and Ferguson, which reported effective PDT treatment when using a 7 mW/cm^2^ red light source [29]. These choices are in line with studies reporting similar efficacy for different irradiances [30] and light doses [22]. With these new irradiation parameters, the light-emitting, fabric-based device has been significantly modified to be more user friendly in terms of dimensions and ergonomics.

This study aims to assess the noninferiority in efficacy at 3 months (primary objective) and superiority in tolerability (secondary objective) of P-PDT compared with C-PDT in the treatment of AK of the forehead and scalp.

Data collection is completed, and data analysis is ongoing. The results are expected in the first half of 2019. In case of a positive assessment, P-PDT could be preferred to the conventionally used C-PDT. Moreover, as P-PDT can be performed in all weather conditions, in any geographic location, and year-round, it could also be preferred to D-PDT. Hence, P-PDT could become the treatment of choice for AK. Furthermore, an ambulatory version of P-PDT could be further investigated.

## Abbreviations

AK: actinic keratosis
ALA: 5-aminolevulinic acid
C-PDT: photodynamic therapy using the conventional protocol
CPP: Comités de Protection des Personnes (the French Ethics Committee)
D-PDT: photodynamic therapy using the daylight European consensus protocol
MAL: methyl aminolevulinate
PDT: photodynamic therapy
P-PDT: photodynamic therapy using the Phosistos protocol
PpIX: protoporphyrin IX
SCC: squamous cell carcinoma
